# The interaction between obesity and sex alters the response to house dust mite in an experimental model of allergic lung inflammation

**DOI:** 10.3389/fphar.2025.1672504

**Published:** 2025-09-22

**Authors:** Amanda Santos-Cavalcante, Alembert Lino Alvarado, Vinicius Cooper Capetini, Julia Camargo Ricci, Gabriel Forato Anhê, Clive Peter Page, Yanira Riffo-Vasquez

**Affiliations:** ^1^ Department of Pharmacology, School of Medical Sciences, State University of Campinas, (UNICAMP) Campinas, Brazil; ^2^ Unit of Pulmonary Pharmacology, School of Cancer and Pharmaceutical Sciences, Institute of Pharmaceutical Science, King’s College London, London, United Kingdom; ^3^ Biology Department, Institute of Bioscience, São Paulo State University (UNESP), São José do Rio Preto, Brazil

**Keywords:** obesity, sex difference, asthma, inflammation, allergy, lungs, females

## Abstract

**Introduction:**

Previous reports have shown that the prevalence of asthma among women is modified by puberty, implying a role for sex hormones in this difference. Additionally, it has been demonstrated that obesity is a significant predisposing factor for asthma, particularly among women. In this regard, populational studies have suggested that severe asthma is more prevalent among obese patients, with worse symptoms, frequent exacerbations and hospitalisations among these patients. This study aimed to elucidate how sex and obesity interact in a murine model of lung allergic inflammation induced by house dust mite (HDM) in male and female animals.

**Methods:**

Male and female C57Bl/6 mice were maintained on a 60% high-fat diet (HFD) or a standard chow diet (SC) for 13 weeks, and on week 11, they underwent an experimental allergic lung inflammation protocol induced by HDM.

**Results:**

Our data showed that, compared to SC-fed male mice, SC-fed female mice exhibit a more severe inflammatory response to HDM exposure. Conversely, the same difference was not observed between HFD female and male mice, with female HFD/HDM mice showing reduced infiltration of leukocytes into the lungs compared to SC/HDM female mice. Similarly, HFD/HDM mice produce lower levels of IgE, IL-5, and IL-13 in the lungs after HDM challenge. However, HFD/Sham female mice displayed notable collagen accumulation in the airways, higher concentrations of SP-D in BAL, and a decrease in the relative gene expression of PECAM-1 in their lungs prior to HDM sensitisation.

**Conclusion:**

Our findings indicate that obesity and sex interact to affect allergic asthma progression in female mice by inducing a pro-inflammatory state in the lung of Sham mice, potentially altering their response to HDM.

## 1 Introduction

According to the World Health Organisation (WHO), in 2022, around 16% of adults older than 18 were obese, which is around 890 million people around the world (World Health Organization, 2020). More concerning, while the percentage of obese children and adolescents in 1990 was only 2% (31 million young people), by 2022, that percentage rose to 8% (160 million young people) (World Health Organization, 2020). Similarly, asthma is a fast-growing chronic disease that affects over 250 million people around the world and is responsible for over 450,000 deaths each year (Venkates and an, 2023). Of particular interest to this study, populational studies have suggested that severe asthma is more prevalent among obese patients, with worse symptoms, more exacerbations and a higher rate of hospitalisations (Lin et al., 2008; Weare-Regales et al., 2024).

The literature strongly corroborates sex differences in asthma (Kole et al., 2024; Fuseini and Newcomb, 2017; Zein and Erzurum, 2015; Leynaert et al., 2012). Some of these studies have suggested a higher asthma distribution in female populations after the onset of puberty, suggesting that sex hormones play a role in this difference (Fuseini and Newcomb, 2017; Zein and Erzurum, 2015; Leynaert et al., 2012). In addition, other studies have demonstrated that obesity is a significant risk factor for asthma, and this is more important in women (Hancox et al., 2005; Chen et al., 2009; Wang et al., 2015) but others have not confirmed this correlation. Furthermore, these sex differences have been demonstrated in experimental animal models. Our group and others have demonstrated that when sensitised and challenged, normal-weight female mice present a more severe lung allergic inflammation and airway remodelling compared to male mice, reflecting what is observed in human asthma (Takeda et al., 2013; e-Lacerda et al., 2020; Blacquiere et al., 2010). However, using a model of obesity, we have also shown in an experimental model of eosinophilic lung inflammation induced by chicken-egg ovalbumin (OVA) that, compared to males, obese female mice exhibit fewer leucocytes in BAL and greater cell density in lung tissue. Additionally, this study demonstrates that obese female mice develop lung remodelling earlier than obese males, suggesting that obesity may modify the sex differences previously reported in this model (e-Lacerda et al., 2020).

Recently, the use of OVA as an allergen in animal models has been increasingly replaced by the use of more clinically relevant allergens, such as HDM(15),pollen (Conejero et al., 2007) or cockroach faeces (Sarpong et al., 2003). It has been suggested that 50%–85% of patients with asthma are typically allergic to *Dermatophagoides sp* and have elevated levels of anti-HDM-specific antibodies (Mostafa et al., 2022). Unlike OVA, HDM induces allergic responses in the airways of mice without the aid of adjuvants and does not induce tolerance, probably due to the presence of proteases in the HDM faecal material (Zhang et al., 2018). In HDM-induced asthma models, elevated Th2 cytokines such as IL-4, IL-5, and IL-13 are consistently observed in the lung and bronchoalveolar lavage fluid (BALF), characteristic of allergic airway inflammation. Other key cytokines, including IL-33, IL-6, and TNF-α, also increase, contributing to eosinophilic inflammation and airway hyperresponsiveness. Th1/Th17 cytokines may also be present depending on the specific model, with some models displaying mixed granulocytic inflammation alongside Th2 responses, airway hyperresponsiveness, antibody production and airways remodelling that shares many features observed in humans with allergic asthma (Gregory et al., 2009; Piyadasa et al., 2016; Kuramoto et al., 2024).

Based on this evidence, the aim of this study was to investigate whether the observations previously made with OVA can be replicated in a more clinically relevant model induced by HDM. To this end, we developed a 13-week obesity model induced by a 60% high-fat diet and created an acute model of allergic lung inflammation induced by HDM in male and female mice.

## 2 Methods

### 2.1 Animals and obesity model

4-week-old male and female C57BL/6 mice were obtained from Envigo, United Kingdom. The animals were separated randomly, and half were fed a high-fat diet (HFD–Bio-Serv, Cat. No. S3282, 60% calories from fat) for 13 weeks. The other half, the SC group, were fed a 5% fat diet over the same duration (SC–Standard Chow, Picolab Rodent Diet 20, Cat: 50503). The effectiveness of the diets was evaluated by measuring a) Weekly body mass for 13 weeks b) Body mass gain over 13 weeks (∆ body mass). c) Mass of white adipose tissue (perigonadal WAT) adjusted by total body mass on week 13.

The experimental procedures adhered to the guidelines stipulated by the Home Office under The Animals (Scientific Procedures) Act (1986) and were approved by the Ethics Committee, King’s College London, study plan SP123803.

### 2.2 HDM sensitisation and challenge

**SCHEME 1:**
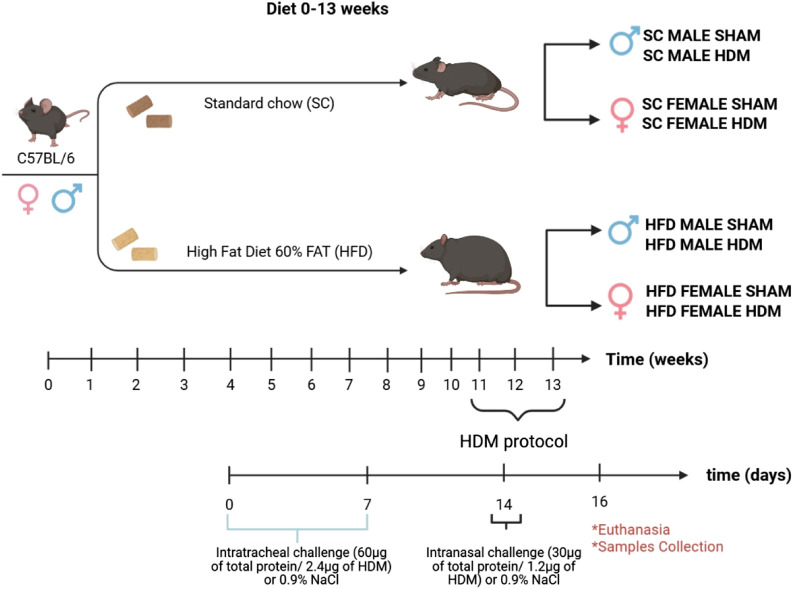
Diet and HDM sensitization scheme.

At the 11th week of the diet (SC and HFD), mice were exposed intratracheally (i.t) to 60 µg of total protein/2.4 µg of Derp-1 in 30 µL of 0.9% NaCl (Day 0; *D. pteronyssinus* extract - Citeq Biologics; Cat. No. 02.01.85), and this procedure was repeated 7 days later (Day 7). Seven days after the second sensitisation (on day 14 of the protocol), an intranasal challenge was performed with 30 µg of total protein/1.2 µg of Derp1 in 30 µL of 0.9% NaCl. Control groups received 30 µL of 0.9% NaCl only (Sham group). The scheme of sensitisation and diet is as below (Scheme 1).

### 2.3 Bronchoalveolar lavage fluid (BALF)

Animals were euthanised 48 h after the last HDM exposure with urethane (2 g/kg i. p.; Sigma Gillingham, United Kingdom). Lungs were lavaged with three aliquots of 0.5 mL of saline through the cannulated trachea. The total number of cells in BALF was manually enumerated with an improved Neubauer haemocytometer. Cytospin preparations of 100 μL of BAL fluid were stained with Diff-Quik (DADE Behring, Germany), and cell populations were enumerated using standard morphological criteria.

### 2.4 Blood collection

Blood was collected through cardiac puncture using the anticoagulant Citrate-dextrose solution (ACD, C3821; Sigma Aldrich). The blood samples were slightly homogenised, and the plasma obtained was stored at −80 °C for further analysis.

### 2.5 Digestion of the lung tissue

The right lobe of the lungs was collected and divided into fragments. One of the fragments was weighed and cut into smaller pieces and placed in RPMI 1640 medium (10 mL, Gibco, United Kingdom), containing Liberase (2 μg/mL, Roche, Germany) and DNAse I Type IV (25 μL/mL, Sigma, United Kingdom). The suspensions were homogenised at 37 °C until the dissociation of the tissue. A lysis buffer was added to remove red cells, and the remaining cells were quantified manually as described above. Total numbers were adjusted by the weight of the tissue in milligrams.

### 2.6 Tissue histology

The left lung lobe (single lobe) was harvested and immersed in 4% formalin for a 72-h fixation period for histological examination. The tissue specimens underwent a systematic processing regimen facilitated by a tissue processing machine (Shandon Citadel 2000 Tissue Processor) and 5 µm sections were cut using a semi-automated microtome (Epredia™ HM 340E).

### 2.7 Histological staining

The following staining techniques were utilised: 1) Haematoxylin and eosin staining (Sigma-Aldrich GHS232 and 318,906, respectively) was employed for a morphological analysis of the peri bronchoalveolar infiltrated area (um^2^); 2) Periodic acid Schiff staining (Schiff-PAS) (Sigma-Aldrich, Cat. Numb. 395B1-KT) was used to measure the % of marked area of goblet cells metaplasia. 3) Masson’s trichrome staining (Abcam–Cat. No. AB150686) was used to measure % of marked area of collagen fibre deposition. All the histological images obtained in this project were taken using a Leica DM 2000 LED microscope with an ×20 or ×40 and then analysed in detail using ImageJ software (Fiji) (https://imagej.nih.gov/ij) or Image-Pro Plus v4.1. We carried out the analysis by randomly selecting the tissue slices and measuring at least five images per sample.

### 2.8 Determination of cytokines, IgE, IgG and estrogen concentrations

IL-4, IL-5, IL-13, IL-17A, IL-33, TNF-α and SP-D concentrations were determined in BAL by multiplex ELISA (R&D # LXSAMSM). CRP, TNF-α and IL-6 concentrations were quantified in plasma by multiplex ELISA (R&D # LXSAMSM). IgE, IgG and Estrogen levels were measured in plasma by conventional ELISA (ThermoFisher Scientific # 88-50460-22; #88-50400-22 and Cayman Chemical CAY501890, respectively), following instructions provided by the manufacturers.

### 2.9 RNA extraction, cDNA preparation and quantitative RT-PCR

Total RNA was extracted from lung samples with the All-in-One Purification Kit (Norgen Biotek Corp., Thorold, Canada) according to the manufacturer’s instructions. The ratio A260 nm/A280 nm was used to evaluate the quality of the RNA. The absorbance was quantified using a NanoDrop ND-1000 Spectrophotometer (Thermo Fisher Scientific, Wilmington, DE, United States), and samples were considered acceptable when absorbance ratios were in the range of 1.9 and 2.1. High-Capacity cDNA Reverse Transcription Kit with RNase Inhibitor (Applied Biosystems, Foster City, United States) were used to reverse transcribe total RNA into cDNA using the T100 Thermal Cycler (Bio-Rad Laboratories, Hercules, United States) following the manufacturer’s instructions. Samples of cDNA were stored at −40 °C.

Samples of cDNA (5 ng/μL) were amplified in the TaqMan Fast Universal master mix (Applied Biosystems, Foster City, United States) with previously tested concentrations of the primer sets for PECAM-1 (*Mm01242576_m1*), Cldn1 (*Mm01342184_m1*), and Ep-CAM (*Mm00493214_m1*). Gene expression was normalized by Eukaryotic 18S rRNA Endogenous Control (VIC™/MGB probe, primer limited) (4319413E). The primer sets were purchased from Applied Biosystems. The amplification was performed on the ViiA 7 Real-Time PCR System (Applied Biosystems, Carlsbad, United States). Negative controls were used in each reaction plate, and all reactions were performed in triplicate. The gene expression was determined according to the 2 (-Delta Delta C(T)) method using the sham group as the calibrator condition.

### 2.10 Statistical analysis

Data analysis was conducted using GraphPad Prism version 10.0.3. Various statistical tests were employed according to the requirements of the experiment, including two-way ANOVA, and three-way ANOVA with Tukey *post-hoc* tests. All the data underwent tests for outlier exclusion utilising the ROUT test with a Q = 1%. The results were expressed as mean ± SEM, and significance was assessed visually, with P-values less than 0.05 being considered statistically significant, denoted as follows: * for p < 0.05, ** for p < 0.01, *** for p < 0.001, and **** for p < 0.0001.

## 3 Results

### 3.1 Obesity model

**FIGURE 1:**
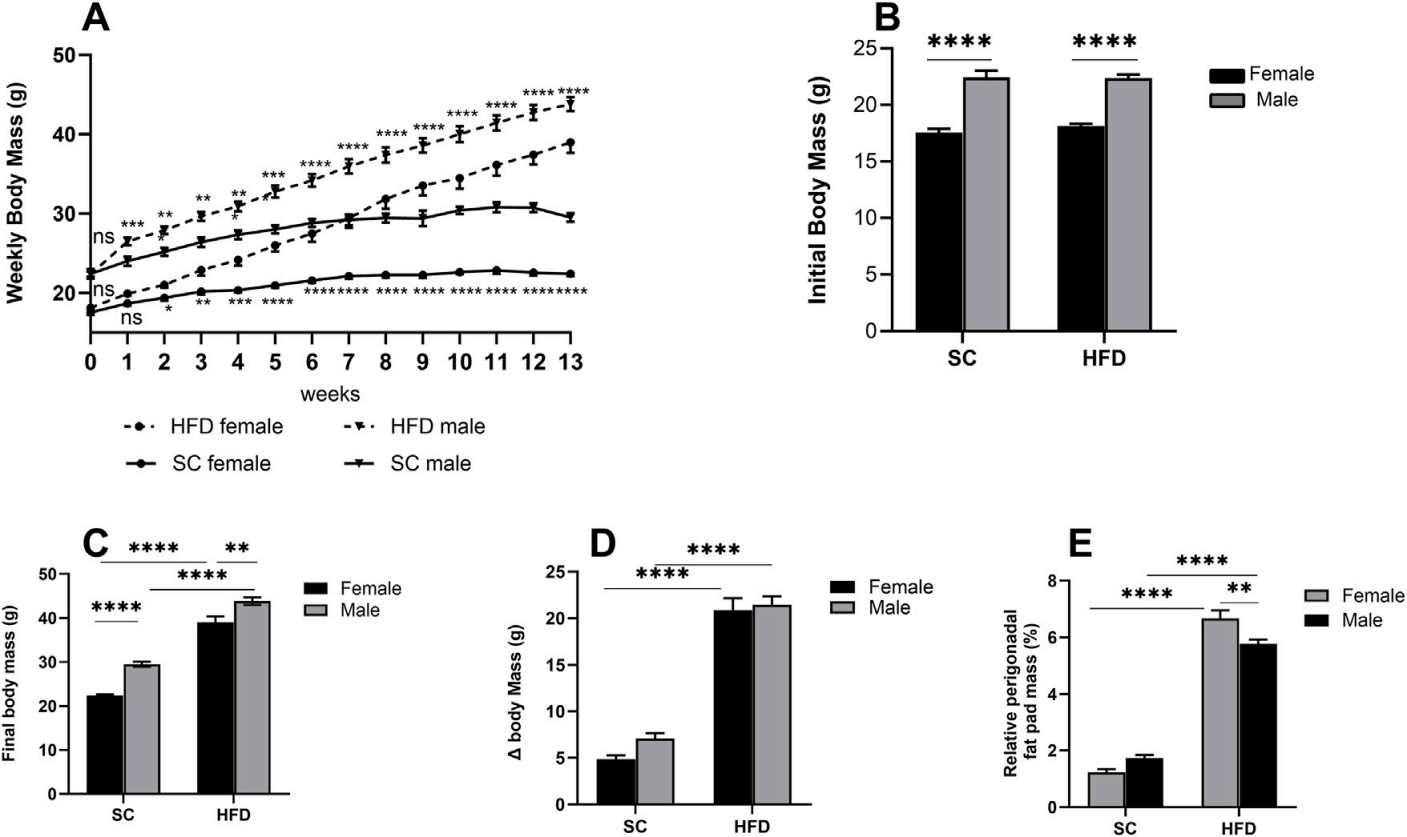
Obesity Model: parameters measured in male and female C57Bl/6 mice fed a SC or HFD diet for 13 weeks. Weekly body mass **(A)**, initial body mass at week 0 **(B)**, final mass at week 13 **(C)**, body mass at week 13 minus mass at week 0 (Δ body mass) **(D)** and relative perigonadal fat pad mass (%) **(E)**. The values presented are expressed as Mean ± SEM for 12 animals in the SC group and 16 animals in the HFD group. By the data distribution, tests for outlier exclusion were performed using the ROUT test with a Q-value of 1%. Subsequently, a two-way ANOVA followed by Tukey’s *post-hoc* test. The differences are visually represented as follows: *p < 0.05; **p < 0.01; ***p < 0.001; ****p < 0.0001.

We have previously characterised 8- and 9-week models of obesity using a high-fat diet (HFD) in mice, demonstrating not only weight gain but also significantly increased levels of cholesterol, leptin, glucose, and triglycerides. In this study, we extended this model to 13 weeks to produce a more pronounced degree of obesity. Before the diet intervention, males were approximately 20% heavier than females of the same age (Figure 1B). After 13 weeks, the 60% HFD led to a 33% increase in body mass in males and a 42.5% increase in females compared to their respective SC-fed controls (p < 0.0001, Figure 1C). When measuring body mass gain (Δ body mass), an increase of around 77% was observed in HFD females compared to SC females, while males showed an increase of about 67% compared to males on a SC diet (p < 0.0001, Figure 1D).

The relative mass of perigonadal fat was also analysed, and after 13 weeks on an HFD, we observed a substantial increase in both males and females (p < 0.0001), with males exhibiting around 70% more gonadal fat and females around 81% more compared to their respective counterparts fed on the SC diet (Figure 1E). When comparing only the groups on an HFD, we found that females have approximately 13% more perigonadal fat than HFD males.

### 3.2 Pulmonary inflammation induced by HDM

#### 3.2.1 Cell count in BALF and lung tissue

**FIGURE 2:**
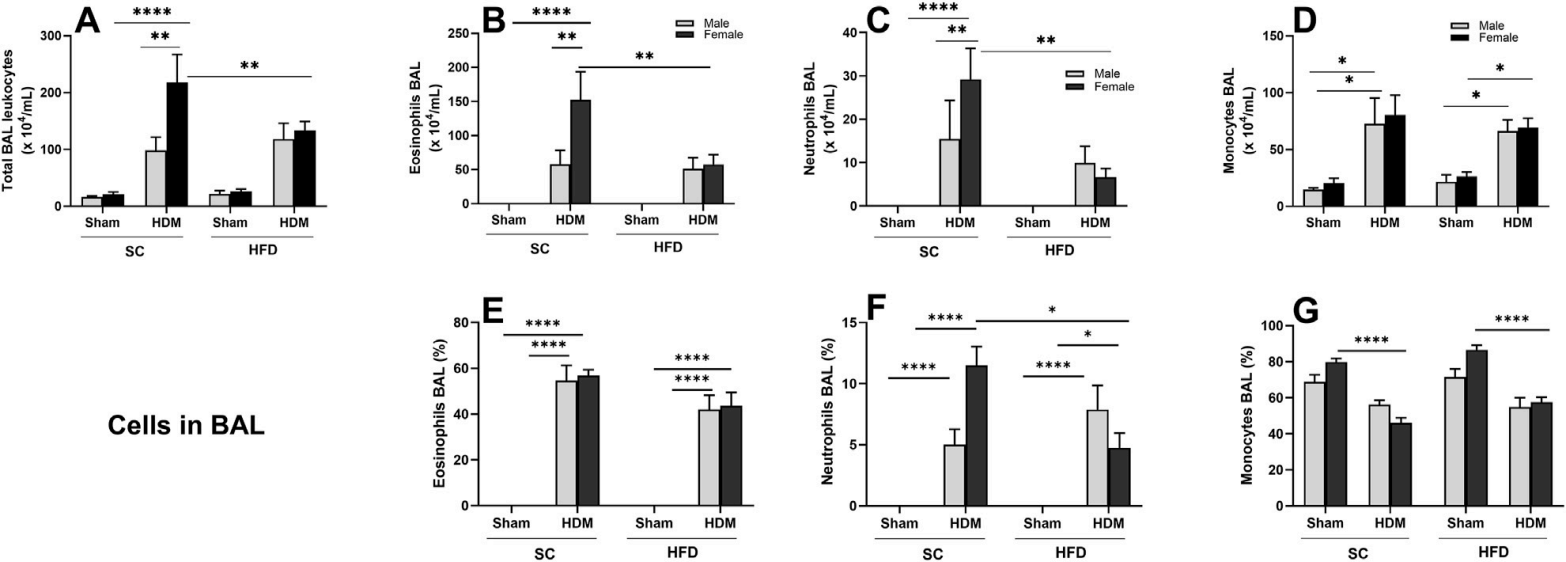
Effect of 13-week SC or HFD diet on total and differential leukocyte count **(A–D)** and percentage of cells **(E–G)** in bronchoalveolar lavage on male and female C57Bl/6 mice challenge with HDM. Total number of Leukocyte **(A)**, Eosinophils **(B,E)**, Neutrophils **(C,F)** and monocytes/macrophages **(D,G)**. The values presented are expressed as Mean ± SEM. In accordance with the data distribution, tests for outlier exclusion were performed using the ROUT test with a Q-value of 1%. Subsequently, a three-way ANOVA followed by Tukey’s *post-hoc* test. Sham animals. N = 6/group for SC animals and N = 8/group for HFD animals. The differences are visually represented as follows: *p < 0.05; **p < 0.01; ****p < 0.0001.

To characterise the inflammation induced by HDM in the lungs of these mice, we quantified the total number and percentage of leukocytes in BALF and lung tissue. The number of leukocytes identified in BALF following the HDM challenge demonstrates that our sensitisation method was effective in inducing allergic inflammation in the lungs (Figure 2A). SC-fed females had a significantly higher total leukocyte count in BALF compared to SC/males and HFD/HDM females when challenged with HDM. We noted a statistically significant rise in the total numbers of eosinophils (Figure 2B) and neutrophils (Figure 2C) in SC female mice in comparison to other groups. Regarding percentage, we only observed a difference between sexes when analysing neutrophil numbers (Figure 2F). The total number of monocytes in BALF in animals exposed to HDM was significantly increased in comparison to sham groups, irrespective of diet or sex, but the reverse was observed in female mice regarding percentage (Figures 2D,G).

**FIGURE 3:**
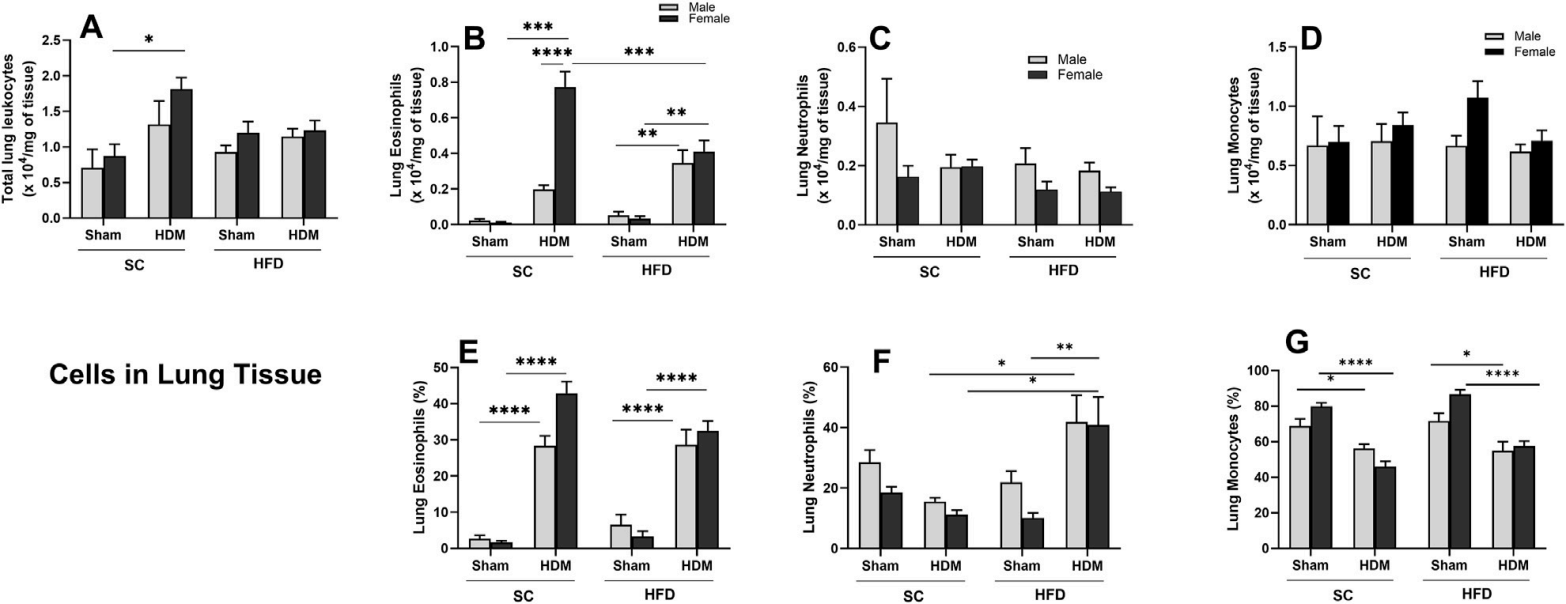
Effect of 13-week SC or HFD diet on total and differential leukocyte count **(A–D)** and percentage of cells **(E–G)** in lung tissue of male and female C57Bl/6 mice challenge with HDM. Total number of Leukocyte **(A)**, Eosinophils **(B,E)**, neutrophils **(C,F)** and monocytes/macrophages **(D,G)**. Total numbers were adjusted by weight of the sample in milligrams. The values presented are expressed as Mean ± SEM. In accordance with the data distribution, tests for outlier exclusion were performed using the ROUT test with a Q-value of 1%. Subsequently, a three-way ANOVA followed by Tukey’s *post-hoc* test. Sham animals. N = 6/group for SC animals and N = 8/group for HFD animals. The differences are visually represented as follows: *p < 0.05; **p < 0.01; ***p < 0.001; ****p < 0.0001.

Female mice displayed a statistically increased total cell number in the lung tissue of SC/HDM groups when compared to sham groups (Figure 3A). The HDM challenge promoted a significant rise in the number and percentage of lung eosinophils in all groups in comparison to sham groups (Figures 3B,E). There is a sex difference in lung tissue regarding the total numbers of eosinophils of SC/HDM animals, with females presenting a more robust response to HDM than males (Figure 3B). We also enumerated a significant increase in the total number of eosinophils in female SC/HDM mice when compared to HFD/HDM females, but this was not observed when analysing percentage increases. The total number of neutrophils in the lung tissue did not change significantly among groups. However, HFD/HDM mice presented a higher percentage of neutrophils in the lung when compared to SC/HDM and HFD/Sham groups and SC/HDM groups (Figures 3C,F). The total number of monocytes remained unchanged among groups (Figure 3D). The percentage increase of monocytes was higher in Sham groups, irrespective of sex or diet (Figure 3G).

#### 3.2.2 Histological analysis of lung tissue

**FIGURE 4:**
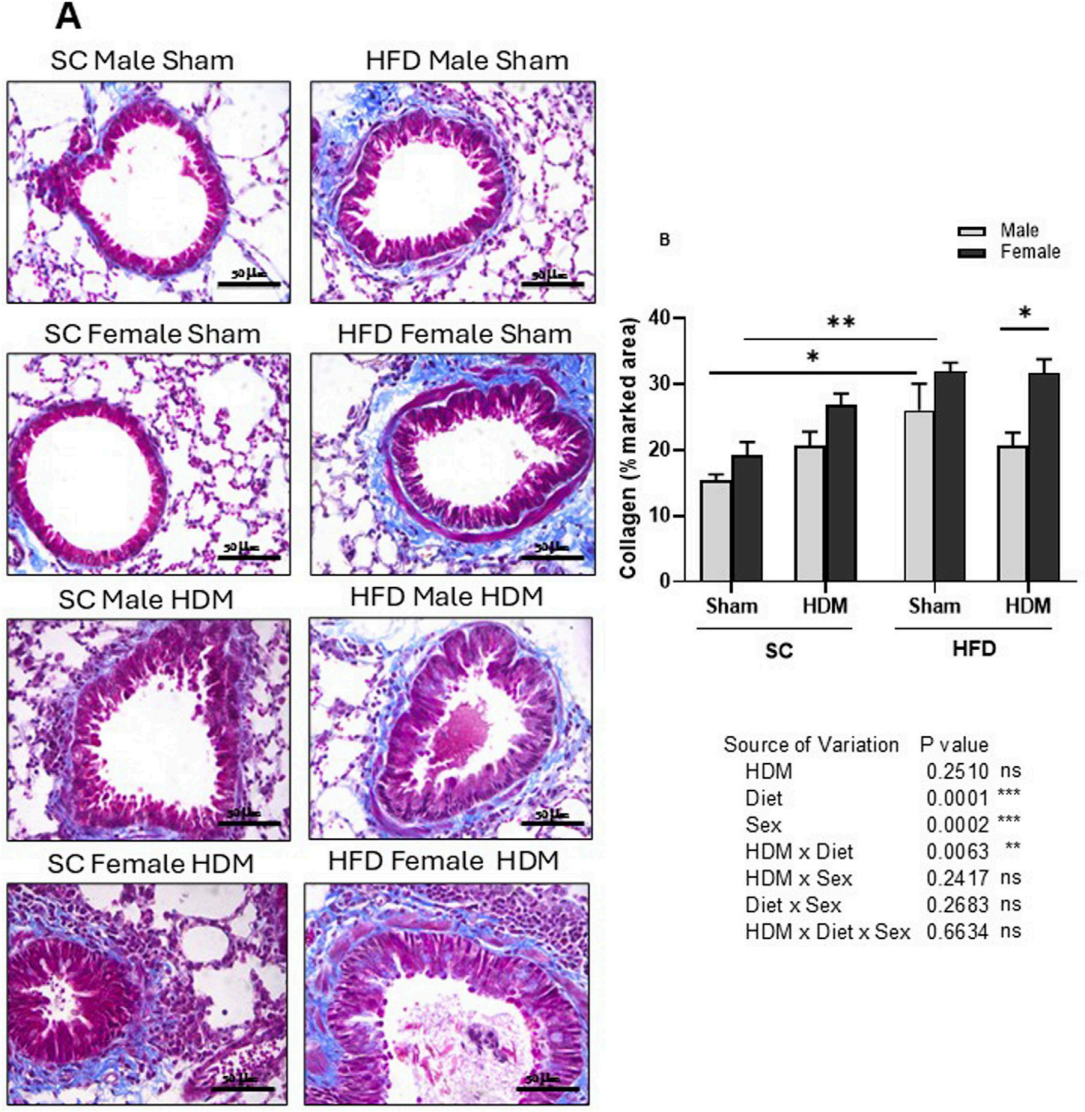
Histological analysis of Masson’s trichrome stained lung tissue of male and female C57Bl/6 mice fed for 13 weeks with SC or HFD diet. Peri bronchial collagen deposition was measured as percentage of marked area analysing 4-6 images per animal. Representative images of all groups **(A)** and quantification of the deposition **(B)**. The values presented are expressed as Mean ± SEM. In accordance with the data distribution, tests for outlier exclusion were performed using the ROUT test with a Q-value of 1%. Subsequently, a three-way ANOVA followed by the Tukey *post-hoc* test. N = 4 mice/group, 4–6 random fields/mouse. The differences are visually represented as follows: *p < 0.01; **p < 0.001.

**FIGURE 5:**
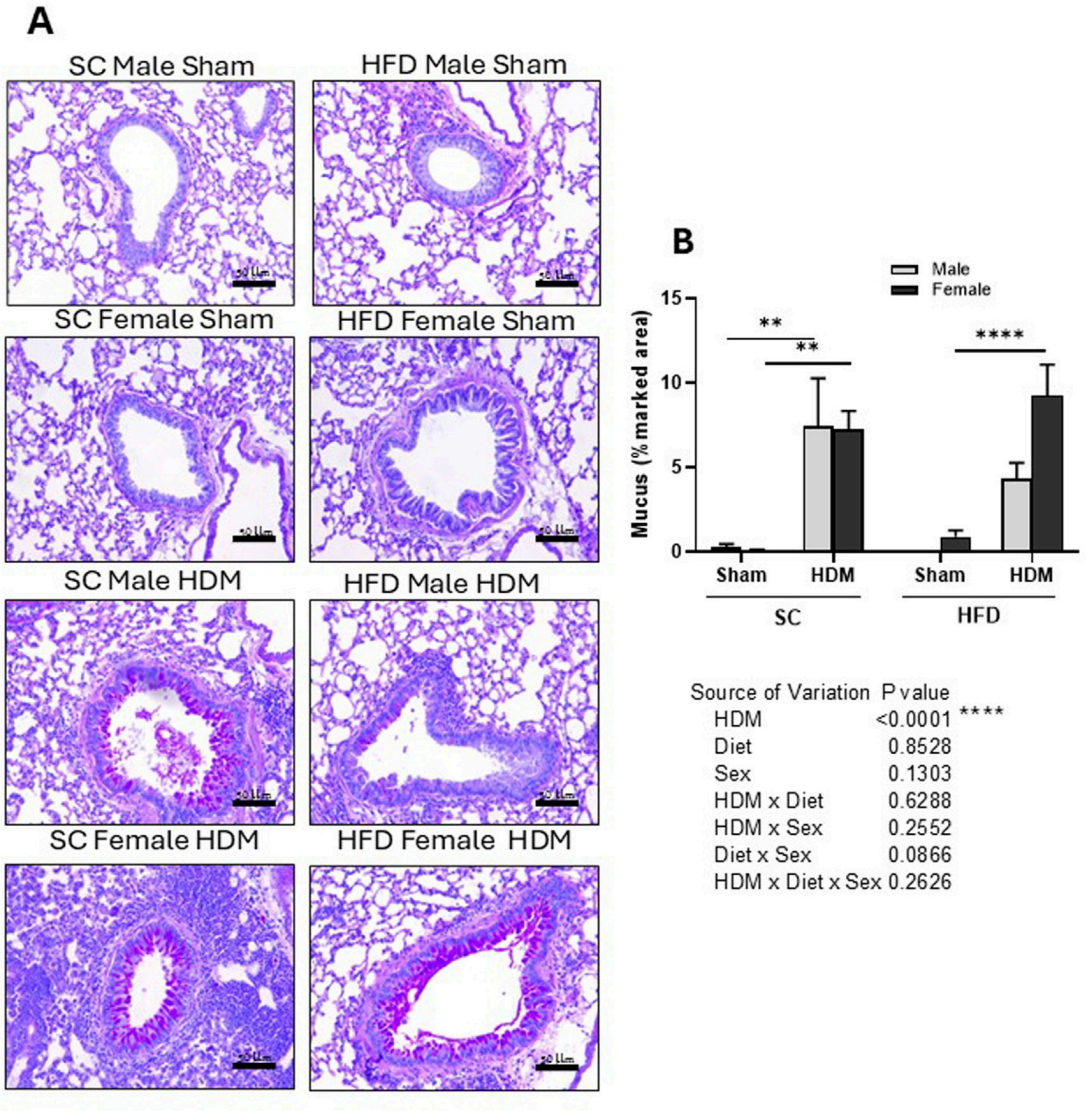
Histological analysis of Periodic acid–Schiff-stained lung tissue of male and female C57Bl/6 mice fed for 13 weeks with SC or HFD diet. Mucus accumulation was measured as percentage of marked area analysing 4–6 images per animal. Representative images of all groups **(A)** and quantification of the deposition **(B)**. The values presented are expressed as Mean ± SEM. In accordance with the data distribution, tests for outlier exclusion were performed using the ROUT test with a Q-value of 1%. Subsequently, a three-way ANOVA followed by the Tukey *post-hoc* test. N = 6-8 mice/group. The differences are visually represented as follows: **p < 0.001; ****p < 0.0001.

To evaluate airway remodelling in our model, we quantified collagen deposition around the airways, goblet cell metaplasia (mucus) and leukocyte accumulation in lung tissue. To this end, tissue sections were stained with Masson trichrome stain (Figure 4), Schiff-PAS staining (Figure 5) or conventional H&E staining (Supplementary Figure S2). Our analysis shows an increased accumulation of collagen around the airways of SC/HDM mice when compared to SC/Sham groups. Interestingly, HFD/Sham mice demonstrated a significantly higher deposition of collagen when compared to SC/Sham groups, which remained higher in HFD/HDM female mice in comparison to males (Figure 4B).

Our analysis also demonstrated that HDM increased the percentage area stained by Schiff-PAS regardless of sex or weight (Figure 5B). However, we did not find statistically significant differences between males or females, although HFD/HDM females presented an increased percentage area of Schiff-PAS staining in comparison to HFD/HDM males (3-way ANOVA).

Lung tissues were also stained with the conventional haematoxylin-eosin (HE) method for morphological analysis. This analysis confirmed results obtained with lung digestion, with SC/HDM female mice presenting a more extensive area of peri-bronchial cell accumulation when compared to the other groups. The results of this staining analysis are in the supplemental section, Supplementary Figure S2.

### 3.3 Levels of circulating IgE and IgG

**FIGURE 6:**
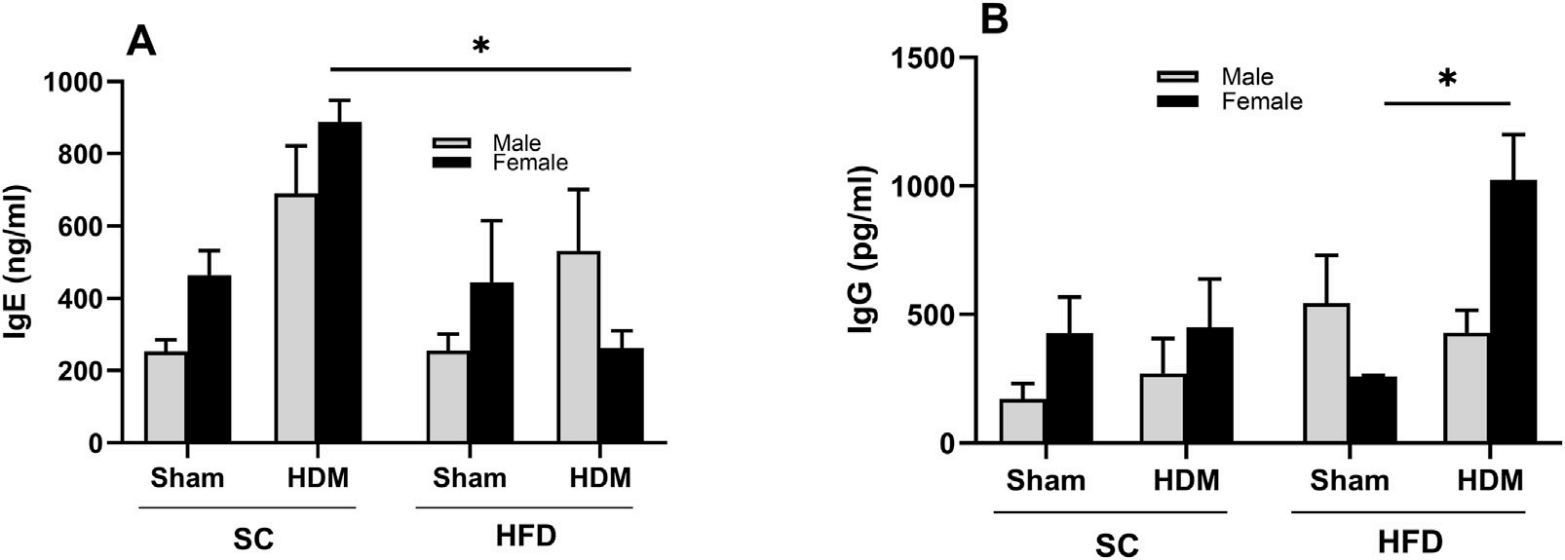
Total levels of circulating IgE **(A)** and IgG **(B)** of male and female C57Bl/6 mice fed for 13 weeks with SC or HFD diet. The values presented are expressed as Mean ± SEM. In accordance with the data distribution, tests for outlier exclusion were performed using the ROUT test with a Q-value of 1%. Subsequently, a three-way ANOVA followed by the Tukey *post-hoc* test. N = 5/group. The differences are visually represented as follows: *p < 0.01.

Allergy to HDM is associated with IgE production (Posa et al., 2017). However, adult onset of obese asthma has been marked by lower IgE levels and infrequent antigen-specific IgE antibody detection (Scott et al., 2016). To evaluate whether this is the case in our model, we analysed the levels of total Th2-related antibody IgE in plasma collected from all groups (Figure 6A). We found increased levels of total IgE in SC mice after HDM challenge, irrespective of sex or weight. However, we did not observe the same increase in HFD/HDM female mice and noted a highly variable response in HFD/HDM male mice (Figure 6A). Analysis of the level of total IgG showed a robust increase in HFD/HDM female mice in comparison to HFD/Sham animals (Figure 6B).

### 3.4 Levels of cytokines in BALF

**FIGURE 7:**
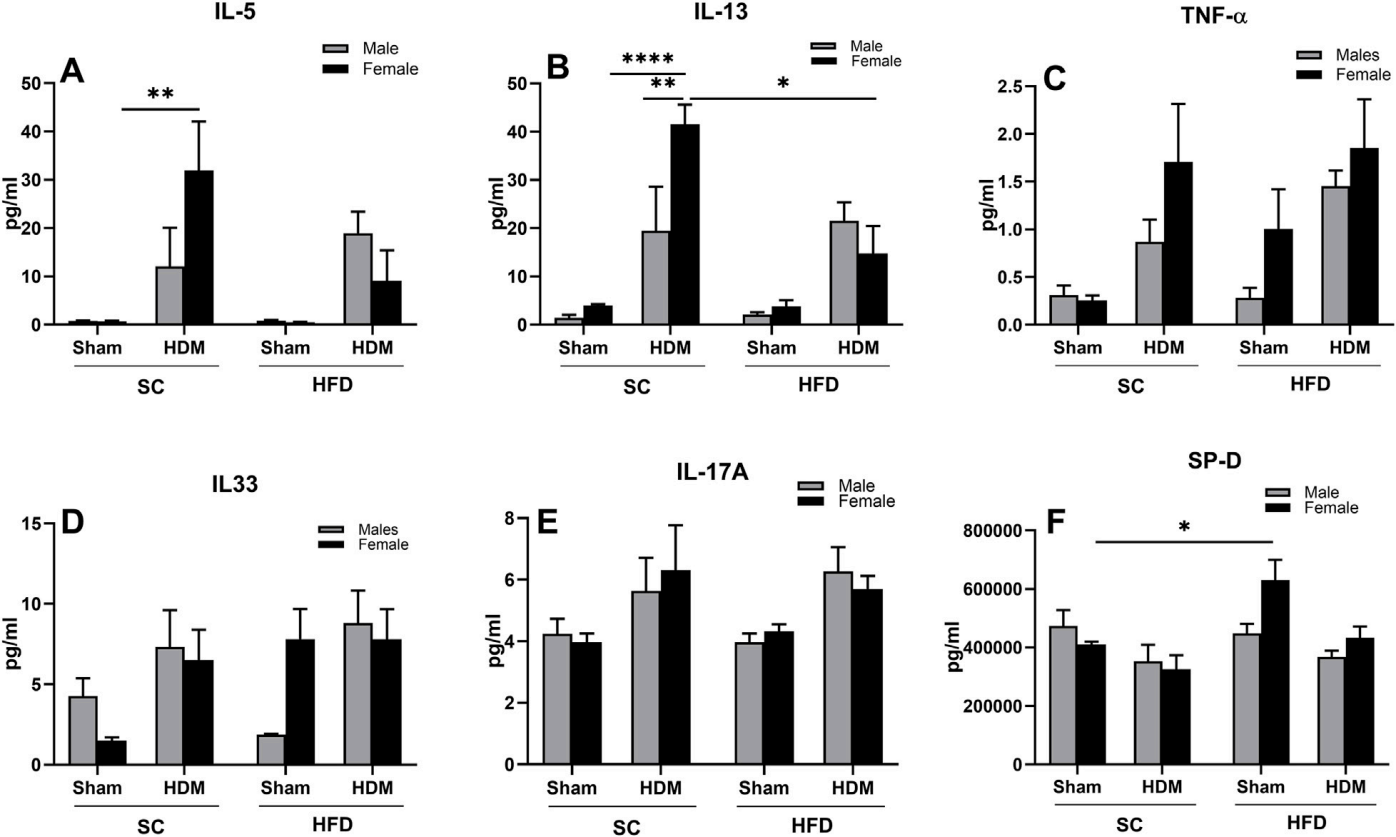
Levels of IL-5 **(A)**, IL-13 **(B)**, TNF-α **(C)**, IL-33 **(D)**, IL-17A **(E)** and SP-D **(F)** in male and female C57Bl/6 mice fed for 13 weeks with SC or HFD diet. The values presented are expressed as Mean ± SEM. In accordance with the data distribution, tests for outlier exclusion were performed using the ROUT test with a Q-value of 1%. Subsequently, a three-way ANOVA followed by the Tukey *post-hoc* test. N = 5/group. The differences are visually represented as follows: *p < 0.01, **p < 0.001.

We also evaluated the release of Th1, Th2 and Th17 cytokines into BAL fluid. We observed that SC/HDM female mice presented a significantly higher concentration of IL-5 in comparison to SC/sham female mice (Figure 7A). Similarly, this group also presented higher level of IL-13 in comparison to SC/HDM males and to HFD/HDM female mice (Figure 7B). HDM female mice had increased levels of TNF-α in comparison to Sham groups, independently of the diet they were fed, although this was not significantly different using a *post-hoc* test (Figure 7C). In addition, HFD/sham female mice showed increased levels of TNF-α in comparison to HFD/sham males and SC/sham groups (3-way ANOVA HDM vs*.* Diet p < 0.5).

Furthermore, HDM mice presented with a non-statistically significant increase in IL-33 when compared to the other groups, irrespective of the diet (Figure 7D). Interestingly, HFD/sham female mice presented higher levels of IL-33 when compared to males or SC/sham females that were not affected by HDM challenge (SC/Sham females: 1.5 ± 0.2 vs. HFD/Sham females 7.8 ± 1.8 pg/mL). Similarly, we did not observe statistical differences among groups regarding levels of IL-17A, but there was a trend towards an increase in HDM mice, irrespective of weight or sex (3 ways ANOVA, allergy factor **p < 0.001), (Figure 7E).

We also measured levels of surfactant protein-D (SP-D), a member of the Collecting family of proteins known to be released in pulmonary inflammation and infections. Our results demonstrate no differences in levels of SP-D between SC/Sham mice and SC/HDM mice in either sex. We did observe however, a significant increase in this surfactant in BAL fluid obtained from HFD/Sham female mice in comparison to SC/Sham mice (Figure 7F).

### 3.5 RT-PCR relative expression of *Claudin-1, Ep-CAM and PECAM-1* in lung tissue of sham mice

**FIGURE 8:**
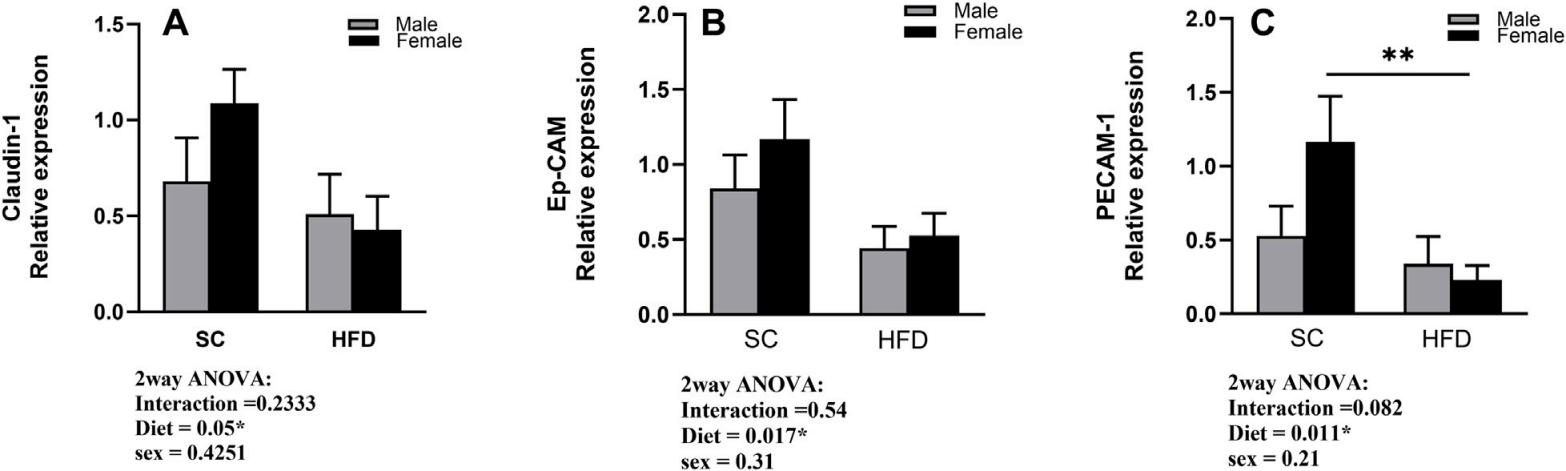
Fold change in gene expression of Claudin-1 **(A)**, Ep-CAM **(B)**, and PECAM-1 **(C)** in the lungs of sham male and female C57Bl/6 mice fed for 13 weeks with SC or HFD. Data were normalised to 18S rRNA and expressed relative to the sham group and are presented as Mean ± SEM. Outliers were identified using the ROUT test with a Q-value of 1%. Statistical analysis was performed using two-way ANOVA followed by Tukey’s post-hoc test. N = 5 for SC groups and N = 8 for HFD groups. Significant differences are indicated as **p < 0 0.01.

Considering our results so far, we then examined the possibility of a pro-inflammatory state of the lungs of obese mice before the sensitisation to HDM. To test this possibility, we analysed the relative expression of the tight junction Claudin-1, the epithelial adhesion molecule, Ep-CAM, and the epithelial/endothelial adhesion molecule, PECAM-1, in the lungs of sham animals using the Real-Time PCR technique. Our results show a decreased expression of Claudin-1 in the lungs obtained from HFD mice when compared to SC sham mice, particularly in females (Figure 8A). Similar changes were also observed when we analysed the expression of the adhesion molecules Ep-CAM and PECAM-1 (Figures 8B,C). Diet was a significant factor in the analysis of the expression of all three proteins (2-way ANOVA), but the *post-hoc* test also showed a significant difference between SC/Sham and HFD/Sham female mice in PECAM-1 expression.

## 4 Discussion

The impact of sex and sex hormones on asthma has been demonstrated in population and experimental studies (Zein and Erzurum, 2015; Scott et al., 2016; Riffo-Vasquez et al., 2007). In childhood, asthma is more common among boys, but this shifts after puberty, with prevalence and severity increasing in girls and adult women (Wijga et al., 2011). Women are more likely to experience more severe, less corticosteroid-responsive subtypes of asthma compared to men (Moore et al., 2010). Some studies have shown that estrogen enhances Th2-mediated airway inflammation, leading to increased eosinophilic infiltration, mucus production, and airway hyperreactivity (Haggerty et al., 2003; de et al., 2007). Similarly, obesity has been identified as a significant modifier of asthma severity, with evidence suggesting that excess adiposity contributes to systemic inflammation, increased airway resistance, and reduced responsiveness to corticosteroid therapy (Peters et al., 2018). However, it remains unclear whether sex differences exist regarding the impact of obesity on asthma. Several studies have suggested that obesity, as a risk factor for asthma, is more important among women (Hancox et al., 2005; Chen et al., 2009; Wang et al., 2015). However, others have not reached the same conclusion, and some have even suggested that obesity worsens asthma symptoms in men but not women (Huovinen et al., 2003; Beuther and Sutherland, 2007). In addition, a survey performed among the US population has found that women have a higher prevalence of the disease only in severe cases of obesity (BMI ≥40) (Wang et al., 2015).

Experimental studies support the association between obesity and asthma (Shore, 2008; Johnston et al., 2007). However, little is known about the relationship between sex and obesity in animal models. Yu and colleagues, using a murine model of OVA and a 7-day challenge protocol, showed no significant differences between sexes, but obese animals demonstrated reduced infiltration of leukocytes into the BAL fluid compared to non-obese mice, regardless of sex (Yu et al., 2018). Our group, also using an acute model of lung allergic inflammation induced by OVA, has demonstrated that, compared to males, obese female mice exhibit fewer leucocytes in BALF and greater cell density in lung tissue (e-Lacerda et al., 2020).

In this study, we have investigated the interaction between sex and obesity in a murine model of allergic airway inflammation induced by HDM. Using an acute sensitisation and challenge protocol, we demonstrate that in animals fed a standard low-fat diet, it is possible to observe clear sex differences in response to this allergen, with females presenting a more robust inflammatory response in comparison to males. This sex difference in response to HDM has been described before in normal-weight animals, so our findings were not unexpected (Blacquiere et al., 2010; Mostafa et al., 2022; Weiss et al., 2021). However, our study has now found that when animals are obese, the inflammatory response to the allergen is altered in female mice, and sex differences are no longer observed in BALF or lung tissue.

Since we and other groups have implicated estrogen in the sex differences observed in lung allergic responses, it is reasonable to consider that obesity may be altering estrogen levels in female mice. To test this hypothesis, we measured the levels of estrogen in the plasma collected from SC and HFD female mice, but we did not find any differences between these two groups (Supplementary Figure S1). This result aligns with one of our previous studies, where we found similar outcomes in a 9-week model of HFD in female mice (Thais Fantozzi et al., 2018). We have therefore ruled out the possibility that the results observed in this study are due to differences in estrogen levels between SC and HFD female mice.

Our group has also previously reported similar findings in a murine model induced by another allergen, OVA. These results demonstrated that allergic obese female mice exhibited a reduced number of leucocytes in the lung lumen when compared to allergic obese male mice, but in contrast, showed a greater accumulation of inflammatory cells in the lung tissue, suggesting that the ability of these cells to cross the airway epithelial barrier may be compromised (e-Lacerda et al., 2020). This hypothesis was supported by a lower expression of adhesion molecules in the lungs of female obese mice (e-Lacerda et al., 2020). In this study, however, whilst we have also observed a reduction in the number of inflammatory cells in BALF, this was not associated with an increased infiltration of inflammatory cells in the lung tissue in comparison to males or SC/HDM females, suggesting a broader effect of obesity on the response to HDM in obese females.

Even though we have not detected a higher infiltration of leukocytes in the lung tissue of HFD/HDM females, we did quantify significantly higher goblet cell metaplasia and accumulation of collagen around the airways in this group and significantly higher levels of SP-D in the BAL of HFD/Sham female mice. Surfactants are related to mucus production in the airways, and in this regard, SP-D, a hydrophilic protein produced in the lung epithelium by alveolar type II cells, is thought to be important in pulmonary immunity and homeostasis, and has been used as a marker of lung integrity (Fernandez-Real et al., 2010). Increased levels of SP-D have been reported in BAL collected from asthmatic patients, and increased production of SP-D has been shown in association with acute lung injury and epithelial activation (McIntosh et al., 1996; Cheng et al., 2000; Peukert et al., 2021). Some studies have reported lower levels of circulating SP-D in obesity, suggesting an impaired innate immunity in these subjects, although very little is known regarding lung levels of SP-D in lavage obtained from obese subjects with asthma (Fernandez-Real et al., 2010; Sorensen et al., 2006). In our model, higher levels of SP-D in BAL of HFD/Sham mice were accompanied by increased deposition of collagen around the airways, along with increased levels of TNF-α, albeit not significant at this time point. In our view, this is an indication of a pro-inflammatory state in the lungs of HFD/sham female mice. This pro-inflammatory state may be related to their altered response to HDM that we are reporting in this study.

Additionally, we have measured an increased level of IL-33 in HFD/Sham female mice. This was not statistically different from SC mice at this specific time point and with this number of samples, but there was a clear trend of an increase in this group. IL-33 is released in the lung by macrophages, dendritic cells, epithelial cells, smooth muscle cells, and innate lymphocytes type-2 (Cayrol, 2021; Schmitz et al., 2005). IL-33 release is usually related to Th2-type responses, but it can also be involved in non-allergic inflammatory responses as an “alarmin” cytokine, signalling the immune system to initiate tissue repair mechanisms (Cayrol, 2021). Pre-formed interleukin 33 is constitutively expressed in the endothelium and epithelial barriers, allowing a rapid response to pathogens, allergens or intrinsic lung injury that will not necessarily result in a Th2-type response (Kearley et al., 2015). In addition, others have reported elevated levels of IL-33 in the serum of obese patients with metabolic disorders, associated with systemic inflammation and cardiovascular diseases (Ta et al., 2021). In our model, we suggest that the release of IL-33 in the lung tissue may also be associated with a pro-inflammatory state in the lungs of HFD/Sham female mice.

Another interesting result of our study is the increased levels of Th2 cytokines and IgE concentrations observed in SC/HDM, but not in HFD female mice. Our results demonstrate lower levels of IL-13, IL-5 and IgE in these animals after HDM challenge, suggesting that their response is not Th2-driven or eosinophil-mediated, as observed in SC mice. On the other hand, we did measure higher levels of circulating IgG in HFD female mice, although we did not detect significantly higher levels of Th1 cytokines at this time point, so we cannot confirm a Th1-driven response to HDM in this group, but we cannot exclude that significant differences may be found at other time points or with higher sample sizes. In addition, HFD mice showed an increased percentage of neutrophils in lung tissue in comparison to SC mice, again suggesting these animals are responding differently to HDM in comparison to SC mice. Supporting our findings, population studies have demonstrated a high number of neutrophils in sputum and blood in obese patients with asthma, and a predominant “non eosinophilic” cluster among obese asthma patients, particularly females (Moore et al., 2010; Holguin et al., 2011). Furthermore, Scott et al. (2011) carried out a study comparing lung inflammation in male and female obese patients with asthma. Their findings demonstrated that the percentage of sputum neutrophils was positively associated with body mass index (BMI) in females with asthma, but not in males, and neutrophilic asthma was present in a higher percentage of female obese patients compared with non-obese subjects. In males, the percentage of neutrophils in sputum was positively associated with fatty acids in plasma but not with BMI. This study did not find a positive correlation between eosinophil numbers and BMI, supporting our observations (Scott et al., 2011).

Since we did not find significantly higher levels of Th1 cytokines in the BALF of HFD mice at this time point, we considered whether these mice presented a Th1-type systemic inflammation instead. To investigate this possibility, we measured plasma levels of the systemic inflammatory markers, C-Reactive Protein (CRP), IL-6 and TNF-α. We detected low levels of TNF-α and IL-6 and very variable levels of CRP with no significant differences among groups. These results suggest that the differences we observe in our model are tissue-specific and not affected by systemic inflammation, but they do not rule out the possibility that a low-grade metabolic inflammation is influencing the lung immunity (Supplementary Figure S3).

In our previous study, we have shown that HFD female mice presented a lower expression of the epithelial adhesion molecule ep-CAM and also the integrin protein CD103, both closely associated with epithelial cell barrier integrity (e-Lacerda et al., 2020). Considering that in this study we observed a lower number of cells in BALF and the lung tissue of HFD female mice, we have investigated the relative gene expression of tight junction protein Claudin-1, ep-CAM and PECAM-1. To further clarify whether the lungs of HFD mice were differentially expressing these molecules before the challenge with HDM, we measured their gene expression in sham animals. Our results show a decreased relative expression for *Claudin-1* and *ep-CAM,* and a significantly lower expression of *PECAM-1* in the lungs obtained from HFD/Sham mice. PECAM-1 (or CD31) is a member of the immunoglobulin superfamily of adhesion molecules. It is a 130-kDa transmembrane protein that is constitutively expressed and can be detected at endothelial cell-cell junctions in the lung (Woodfin et al., 2007). Some studies have proposed that PECAM-1 participates in the preservation of vascular integrity in homeostasis, as well as contributing to the restoration of vascular integrity following barrier disruption and is implicated in trans endothelial leukocyte migration (Muller et al., 1993; Privratsky et al., 2011). In our study, the gene expression of this adhesion molecule is significantly suppressed in HFD/Sham mice, which may contribute to the lower number of leukocytes in the lung tissue after HDM challenge. Supporting this hypothesis, we have enumerated significantly higher numbers of total leukocytes and platelets in the circulation of HFD/HDM female mice in comparison to SC/HDM female mice (Supplemental section, Supplementary Figure S4), strongly suggesting not only an impaired leukocyte epithelial crossing (Claudin-1 and ep-CAM), but also through the endothelial barrier (claudin-1 and PECAM-1). Further studies are necessary to elucidate the mechanism underlying this impairment.

## 5 Conclusion

In summary, our findings indicate that obesity and sex interact to affect allergic asthma progression in female mice by inducing a pro-inflammatory state in the lung, potentially changing their reaction to HDM. This pro-inflammatory state is not dependent on changes in circulating levels of estrogen or systemic inflammation. Further studies are necessary to determine the underlying mechanisms of these altered responses to HDM observed in obese female mice. This study may contribute to the understanding of the clinical differences observed in the incidence, development and severity of asthma in obese subjects, especially in female patients.

## Data Availability

The datasets presented in this study can be found in online repositories. The names of the repository/repositories and accession number(s) can be found in the article/Supplementary Material.

## Abbreviations

HFD, High fat diet; SC, Standard chow diet; HDM, House dust mite; OVA, Chicken egg-ovalbumin.
