# Supplementary material for: The interaction between obesity and sex alters the response to house dust mite in an experimental model of allergic lung inflammation
Source: Front Pharmacol. 2025 Sep 22;16:1672504. doi: 10.3389/fphar.2025.1672504 (PMC12498017; doi:10.3389/fphar.2025.1672504)
Supplement: Supplementary file 1 [file Supplementaryfile1.docx]

**Supplemental Figures**

**S1:** **Effect of 13-week SC or HFD diet on** **estrogen levels in plasma of female C57Bl/6 mice.** The values presented are expressed as Mean ± SEM. In accordance with the data distribution, tests for outlier exclusion were performed using the ROUT test with a Q-value of 1%. Subsequently, a 2-way ANOVA followed by Tukey’s *post-hoc* test. Sham animals. N=10 for SC animals and N=9 for HFD animals.

**
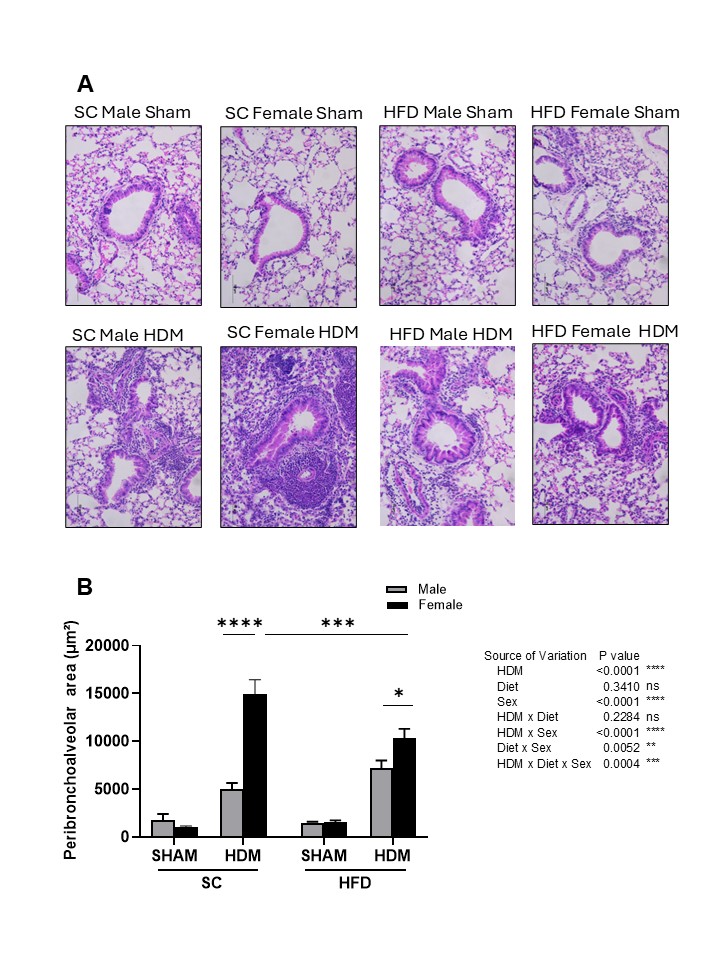
**

**S2: Histological analysis of** **Haematoxylin and Eosin-stained lung tissue of** **male and female C57Bl/6 mice fed for 13 weeks with SC or HFD diet.** Representative images show the leukocyte accumulation around the airways (A). Peribronchial inflammatory cell infiltrate was measured in µm^2^ analysing 3 images per animal (B). The values presented are expressed as Mean ± SEM. In accordance with the data distribution, tests for outlier exclusion were performed using the ROUT test with a Q-value of 1%. Subsequently, a three-way ANOVA (Factor one: diet; Factor two: sex and factor three: HDM exposure) e followed by the Tukey *post-hoc* test was conducted for analysis. Sham animals: SC=6/group, HFD=8/group The differences are visually represented as follows: *p < 0.05; **p < 0.01; ***p <0.001; ****p < 0.0001.

**S3.** **Levels of CRP (A), TNF-α (B) and IL-6 (C) measured in plasma of male and female C57Bl/6 mice fed for 13 weeks with SC or HFD diet.** The values presented are expressed as Mean ± SEM. In accordance with the data distribution, tests for outlier exclusion were performed using the ROUT test with a Q-value of 1%. Subsequently, a three-way ANOVA followed by the Tukey *post-hoc* test. N=5/group.

**S4. Effect of 13-week SC or HFD diet on total leukocyte (A) and platelets count (B) in blood on male and female C57Bl/6 mice challenge with HDM.** The values presented are expressed as Mean ± SEM. In accordance with the data distribution, tests for outlier exclusion were performed using the ROUT test with a Q-value of 1%. Subsequently, a three-way ANOVA followed by Tukey’s *post-hoc* test. Sham animals. N=6/group for SC animals and N=8/group for HFD animals. The differences are visually represented as follows: *p < 0.01; **p < 0.001
